# AI-based body composition analysis of CT data has the potential to predict disease course in patients with multiple myeloma

**DOI:** 10.1038/s41598-025-11560-3

**Published:** 2025-07-21

**Authors:** Franz Wegner, Malte Maria Sieren, Hanna Grasshoff, Lennart Berkel, Christoph Rowold, Marcel Philipp Röttgerding, Soleiman Khalil, Sam Mogadas, Felix Nensa, René Hosch, Gabriela Riemekasten, Anna Franziska Hamm, Nikolas von Bubnoff, Jörg Barkhausen, Roman Kloeckner, Cyrus Khandanpour, Theo Leitner

**Affiliations:** 1 Institute of Interventional Radiology, University Hospital Schleswig-Holstein, Lübeck, Germany; 2 Fraunhofer Research Institution for Individualized and Cell-Based Medical Engineering, Fraunhofer IMTE, Lübeck, Germany; 3 Institute of Radiology and Nuclear Medicine, University Hospital Schleswig-Holstein, Lübeck, Germany; 4 Clinic of Rheumatology and Clinical Immunology, University Hospital Schleswig-Holstein, Lübeck, Germany; 5 Department of Hematology and Oncology, University Cancer Center Schleswig-Holstein, University Hospital Schleswig-Holstein, University of Lübeck, Lübeck, Germany; 6 Institute of Diagnostic and Interventional Radiology and Neuroradiology, University Hospital Essen, Essen, Germany; 7 Institute for Artificial Intelligence in Medicine, University Hospital Essen, Essen, Germany

**Keywords:** Bone cancer, Cancer epidemiology, Cancer imaging, Tumour heterogeneity

## Abstract

The aim of this study was to evaluate the benefit of a volumetric AI-based body composition analysis (BCA) algorithm in multiple myeloma (MM). Therefore, a retrospective monocentric cohort of 91 MM patients was analyzed. The BCA algorithm, powered by a convolutional neural network, quantified tissue compartments and bone density based on routine CT scans. Correlations between BCA data and demographic/clinical parameters were investigated. BCA-endotypes were identified and survival rates were compared between BCA-derived patient clusters. Patients with high-risk cytogenetics exhibited elevated cardiac marker index values. Across Revised-International Staging System (R-ISS) categories, BCA parameters did not show significant differences. However, both subcutaneous and total adipose tissue volumes were significantly lower in patients with progressive disease or death during follow-up compared to patients without progression. Cluster analysis revealed two distinct BCA-endotypes, with one group displaying significantly better survival. Furthermore, a combined model composed of clinical parameters and BCA data demonstrated a higher predictive capability for disease progression compared to models based solely on high-risk cytogenetics or R-ISS. These findings underscore the potential of BCA to improve patient stratification and refining prognostic models in MM.

**Supplementary Information:**

The online version contains supplementary material available at 10.1038/s41598-025-11560-3.

## Introduction

Multiple myeloma (MM) is a chronic malignant disease of the bone marrow. As the prognosis of MM patients is heterogenous, predictive markers are of high clinical importance to allow for early risk stratification. The Revised-International Staging System (R-ISS) and high-risk cytogenetics have been established as valid prognostic factors^1,2^. Nevertheless, disease progression is still difficult to anticipate in clinical routine and additional prognostic markers are needed.

Body composition analysis (BCA) is a promising tool for the prediction of disease trajectories and survival in chronic diseases. In the past, Dual-Energy-X-ray Absorptiometry (DEXA) and body impedance measurements were the gold standard for BCA. The main disadvantage of these time-consuming methods is the added workload and complexity they introduce to the routine examinations performed in MM patients. In the last years, computer tomography (CT)-derived BCA has become available and has shown very promising results for a several chronic and malignant diseases^3–6^. In the existing studies, BCA parameter extraction was realized by manually or semi-automatically performed segmentations on defined anatomical levels. These values provided estimates of the fat and muscle mass for the whole body^7^.

Recently, artificial intelligence (AI) was introduced to BCA^8^. In contrast to manual segmentation-based BCA approaches, AI-based BCA enables completely automated and volumetric data analysis of specific tissue compartments. Images from routinely performed CT scans are fully suitable for AI-based BCA. This technique allows for a time- and cost-effective generation of precise and reproducible BCA results.

So far, only a few studies investigated the association between body composition and outcome in patients with MM. For example, obesity was identified as negative predictive parameter for survival^9^. Nevertheless, the clinical value of CT-based BCA for MM patients remains unclear, as the scarce existing literature offers heterogenous results^10–12^.

The aim of this study was to investigate the potential of patient stratification by AI-based volumetric BCA in MM. Therefore, the correlations of BCA-based quantitative imaging biomarkers with clinical parameters were studied, BCA endotypes were identified and the survival rates of identified patient clusters were analyzed.

## Materials/subjects and methods

### Patient recruitment

A total of 91 patients were included in this study. Ethics approval was given, and informed consent was waived by the “Ethics Committee of the University of Luebeck”, reference number 2024-140_1, 30.04.2024 / IK. All methods in this study were performed in accordance with relevant guidelines and regulations. All patients were first diagnosed MM between January 2018 and December 2023 at a single-centre university hospital. MM was diagnosed according to the revised International Myeloma Working Group (IMWG) criteria^13^. At least one whole-body CT scan was required for inclusion in this study. Patients were retrospectively assessed for basic demographics, laboratory values and treatment modalities. Risk stratification for disease progression was performed by determining the R-ISS score and the presence of high-risk cytogenetics, defined as translocations t(4;14), t(14;16) or del(17p)^14^. Progression-free survival and overall survival was assessed from the start of treatment to the last follow-up within the study period. Of the 91 patients included in this analysis, the most frequently administered first-line therapy regimen was VD (bortezomib and dexamethasone, 20.9%), followed by VCD (bortezomib, cyclophosphamide, dexamethasone, 18.7%) and Dara-VTD (daratumumab, bortezomib, thalidomide, dexamethasone, 15.4%). 39 patients underwent autologous stem cell transplantation (ASCT) (42,9%). An overview of the different therapeutic regimes in this cohort is given in Supplementary Table 1.

### CT imaging

CT low-dose scans were performed during the two months after the initial diagnosis of MM to determine the status of osseous (osteolytic) manifestations in the beginning of the disease course. All included patients underwent whole body imaging with the clinical CT scanners Philips Spectral CT 7500 (Philips, The Best, Netherlands) and Siemens Somatom Definition AS (Siemens, Erlangen, Germany). The scans were performed without application of contrast agent. The standard scan parameters for the Philips Scanner were the following: tube voltage = 120 kV, slice thickness = 1 mm, total collimation width = 80, pitch factor = 0.7. The scan parameters in the Siemens CT were: tube voltage = 120 kV, slice thickness = 1 mm, total collimation width = 38.4, pitch factor = 1.5. The tube current was automatically adapted in both scanners.

### Body composition analysis

The CT images of the whole-body scans were processed via a BCA algorithm, which is based on a convolutional neural network^8^. By means of automatically performed segmentations, this algorithm volumetrically quantifies tissue compartments and bone density. The values are given as mean per slice in ml. The following body composition parameters were extracted from the CT data: total adipose tissue (TAT), visceral adipose tissue (VAT), subcutaneous adipose tissue (SAT), intramuscular adipose tissue (IMAT), epicardial adipose tissue (EAT), pericardial adipose tissue (PAT), skeletal muscle volume (muscle), and bone mineral density (bone). Additionally, we calculated the cardiac marker (CM) index. The CM was computed by dividing the sum of PAT and EAT by muscle mass. It represents the relationship between epicardial and pericardial fat depots^15^. Furthermore, the sarcopenia marker (SM) was computed as the ratio of muscle mass to the combined total of intra- and intermuscular adipose tissue and bone^15^.

### Statistics

The statistical analyses were carried out by using the software R version 4.4.2 and Jamovi version 2.6.19. Normal distribution of BCA-parameters was analyzed using Shapiro–Wilk normality test. BCA parameters were compared according to high-risk cytogenetics, sex and outcome (disease progression vs. progression free survival) using Mann–Whitney *U* test. The comparison of the BCA parameters according to R-ISS score was performed using Kruskal–Wallis test with Dunn’s post hoc test. The correlation analysis between the BCA parameters as well as age was performed determining the Spearman correlation coefficient. As these analyses revealed significant differences in BCA parameters according to sex and significant correlations of BCA parameters with age, further analyses were performed adjusting the data for age and sex. To enable an adjustment of data, BCA parameters were normalized using the R package ‘*bestNormalize’*. Subsequently, the distribution of the normalized BCA data was tested by determining skewness, kurtosis and the Shapiro–Wilk normality test. Identification of BCA endotypes was performed using k-means cluster analysis, applied to the full set of normalized parameters. The optimal number of clusters was identified using the silhouette method. To visualize the separation of the identified clusters and the contribution of individual BCA variables, a Principal Component Analysis (PCA) biplot was generated. Importantly, PCA was used exclusively for visualization purposes and was not part of the clustering process. The survival rates of both clusters were compared by applying a Log-rank test.

The stepwise model selection was performed by using the packages ‘*VIM’*, ‘*MASS’*, ‘*pROC’* and, ‘*ggplot2’*. Missing values were primarily imputed by using k-nearest neighbours method provided by the *VIM* package (k = 5). Four logistic regression models were developed to predict the outcome of the patients based on the following parameter groups:

1. A model based on the presence of high-risk cytogenetics.
2. A model incorporating the R-ISS score.
3. A BCA model employing the parameters: bone density, muscle volume, IMAT, TAT, SAT, VAT, PAT, EAT, SM and CM.
4. A combined model employing the BCA parameters and the clinical parameters (high-risk cytogenetics, R-ISS, sex, age).

A stepwise model selection approach using the Akaike Information Criterion (AIC) was employed to refine the BCA model and the combined model using the ‘*MASS’* package. The AIC is a quality measure parameter for the model evaluation. Receiver Operating Characteristic (ROC) curves were generated to evaluate the predictive performance of each of the four models. Additionally, the Area Under the Curve (AUC) was calculated for the models. The ROC curves for the models were visualized using the ‘*ggroc’* function of the ‘*pROC’* package. Statistical significance was set to a *p*-value < 0.05 in all analyses.

## Results

### Demographic and clinical characterisation of the study cohort

Within the cohort of 91 patients, a male predominance was observed (58.2%, 53/91) (Table 1). The mean age at CT examination was 65.9 ± 10.9 years. Most patients had an R-ISS score of 2 (53.2%, 41/77). A high-risk cytogenetic profile was observed in 16.0% (13 out of 81) of all patients. The mean clinical follow-up time was 15.7 ± 15.5 month (median: 9.2, IQR: 13.3).

**Table 1:** The table summarizes the demographics, clinical and BCA data of 91 MM patients. Demographically, the group is predominantly male with a mean age of 65.9 years.

Parameter	Total (N = 91)
*Demographic parameters*	
Sex	
Female: *N (%), n*_*total*_	38 (41.8%), 91
Male: *N (%), n*_*total*_	53 (58.2%), 91
Age (years): *M* ± *SD, n*_*total*_	65.9 ± 10.9, 91
*Disease characteristics*	
R-ISS	
1: *N (%),n*_*total*_	14 (18.2%), 77
2: *N (%),n*_*total*_	41 (53.2%), 77
3: *N (%),n*_*total*_	22 (28.6%), 77
High-risk cytogenetics: *N (%), n*_*total*_	13 (16.0%), 81
Body composition parameter	(mean per slice)
Bone density (HU): *M* ± *SD, n*_*total*_	215.2 ± 38.8, 91
Skeletal muscle volume (ml): *M* ± *SD, n*_*total*_	19.0 ± 10.7, 91
IMAT (%): *M* ± *SD, n*_*total*_	6.1 ± 4.9, 91
TAT (ml): *M* ± *SD, n*_*total*_	39.2 ± 29.5, 91
VAT (ml): *M* ± *SD, n*_*total*_	9.0 ± 7.5, 91
SAT (ml): *M* ± *SD, n*_*total*_	23.2 ± 18.1, 91
PAP (ml): *M* ± *SD, n*_*total*_	0.6 ± 0.5, 91
EAT (ml): *M* ± *SD, n*_*total*_	0.3 ± 0.2, 91
CM: *M* ± *SD, n*_*total*_	0.05 ± 0.03, 91
SM: *M* ± *SD, n*_*total*_	0.09 ± 0.04, 91

Genetic analysis of the patients revealed high-risk cytogenetics in 16.0%. The majority of the patients had an R-ISS of 2 (53.2%), followed by 3 (28.6%) and 1 (18.2%).

The mean body composition parameters ± standard deviation of the cohort are depicted in Table 1. The correlation analysis revealed weak to moderate correlations between age and bone density (Spearman r −0.299, *p* = 0.004), EAT (Spearman r 0.211, *p* = 0.044) and CM (Spearman r 0.308, *p* = 0.003) (Supplementary Fig. 1A). Moreover, a significant positive correlation was observed between all adipose tissue compartments, as well as between adipose tissue compartments and CM and SM. In addition, the muscle volume correlated positively with TAT, IMAT, SAT, VAT, PAT, EAT, and SM. Furthermore, bone density correlated positively with muscle and negatively with CM. In addition, a Mann–Whitney *U* analysis revealed a significant difference between male and female study participants regarding muscle volume (W = 549, *p* = 0.0002), VAT (W = 611, *p* = 0.0015), PAT (W = 515, *p* = 0.0001), EAT (W = 691, *p* = 0.011), CM (W = 746, *p* = 0.036) and SM (W = 592, *p* = 0.0009) (Supplementary Fig. 1B).

### Cardiac marker index differs between patients depending on high-risk cytogenetics

**Fig. 1:**
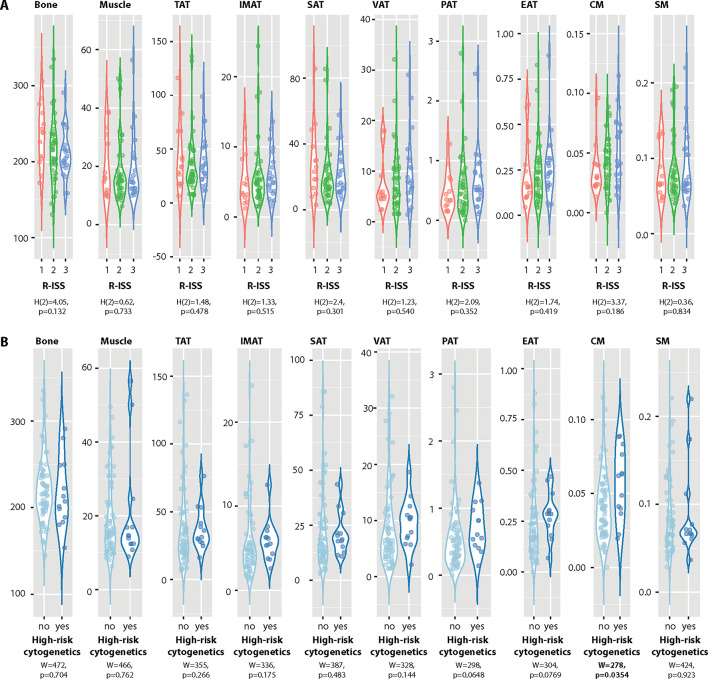
Violin plots illustrating BCA parameters in patients stratified by (**A**) R-ISS scores (1, 2, or 3) and (**B**) the presence or absence of high-risk cytogenetics (no/yes). Comparisons among R-ISS categories were performed using the Kruskal–Wallis test, while differences based on cytogenetic risk were analyzed with the Mann–Whitney U test. Statistical test results are presented within the figure.

The BCA parameters were analyzed across R-ISS categories, with the results presented in Fig. 1A. No significant differences in BCA parameters were identified among the three R-ISS categories. Additionally, a comparison of BCA parameters was conducted between patients with and without high-risk cytogenetics using the Mann–Whitney *U* test, as shown in Fig. 1B. Patients with high-risk cytogenetic profiles exhibited a significantly elevated cardiac marker index compared to those without high-risk cytogenetics (W = 278, *p* = 0.0354). No statistically significant differences were observed for the remaining BCA parameters between the two groups.

### Distinct Body composition parameters differ between patients with progression-free survival and progression

Clinical follow-up data were available for all patients. Patients were followed-up for a mean duration of 15.7 ± 15.5 months (minimum follow-up 0.6 months, maximum 68.8 months). Progression-free survival was observed in 48 (52.7%) patients, whereas 43 (47.3%) patients developed disease progression. Disease progression was defined according to the response criteria established by the International Myeloma Working Group (IMWG) which was observed in 32 (35.2%) of patients, or death of all causes in 11 (12.1%) patients.

**Fig. 2:**
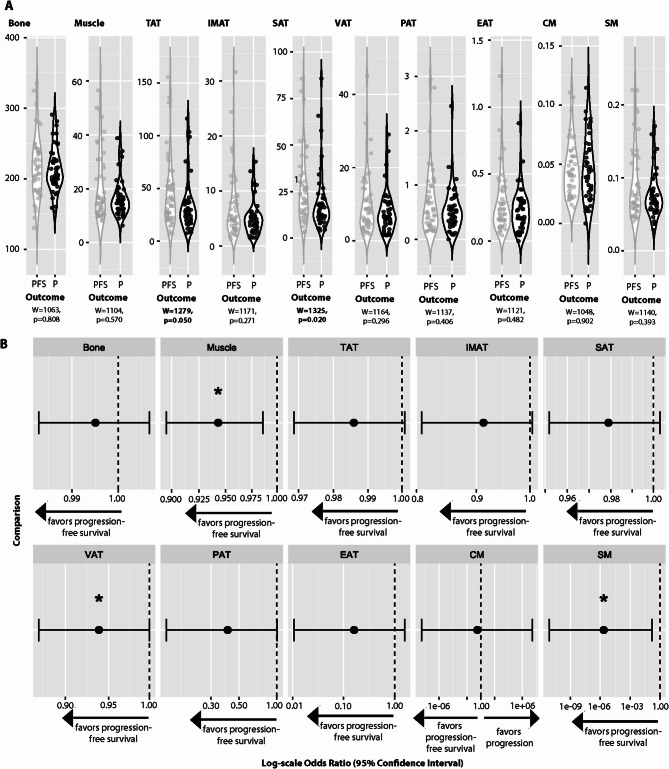
Distinct body composition parameters differ between patients with progression-free survival and those with disease progression or death during follow-up. (**A**) Violin plots comparing BCA parameters between patients with progression-free survival (grey) and those with disease progression or death during follow-up (black), analyzed using the Mann–Whitney U test. (**B**) Forest plots showing the results of binomial logistic regression assessing the association between progression-free survival and disease progression or death during follow-up, adjusted for age and sex. Odds ratios with 95% confidence intervals are displayed on a logarithmic scale, with significant results (confidence intervals not crossing 1) highlighted by a star.

In patients who experienced disease progression or died during follow-up, the volume of subcutaneous adipose tissue (Mann–Whitney *U* test, W = 1325, *p* = 0.020) was significantly lower compared to those with progression-free long-term follow-up (Fig. 2A). The volume of total adipose tissue showed a trend to be lower in patients of the progression/death during follow-up group (Mann–Whitney *U* test, W = 1279, *p* = 0.05). Binomial logistic regression revealed an association with lower muscle volume, lower visceral adipose tissue and lower sarcopenia marker and progression-free survival. The data were adjusted to age and sex. The respective results are depicted in Fig. 2B.

### Clustering of patients based on BCA parameters identified groups with significantly different disease progression probabilities

**Fig. 3:**
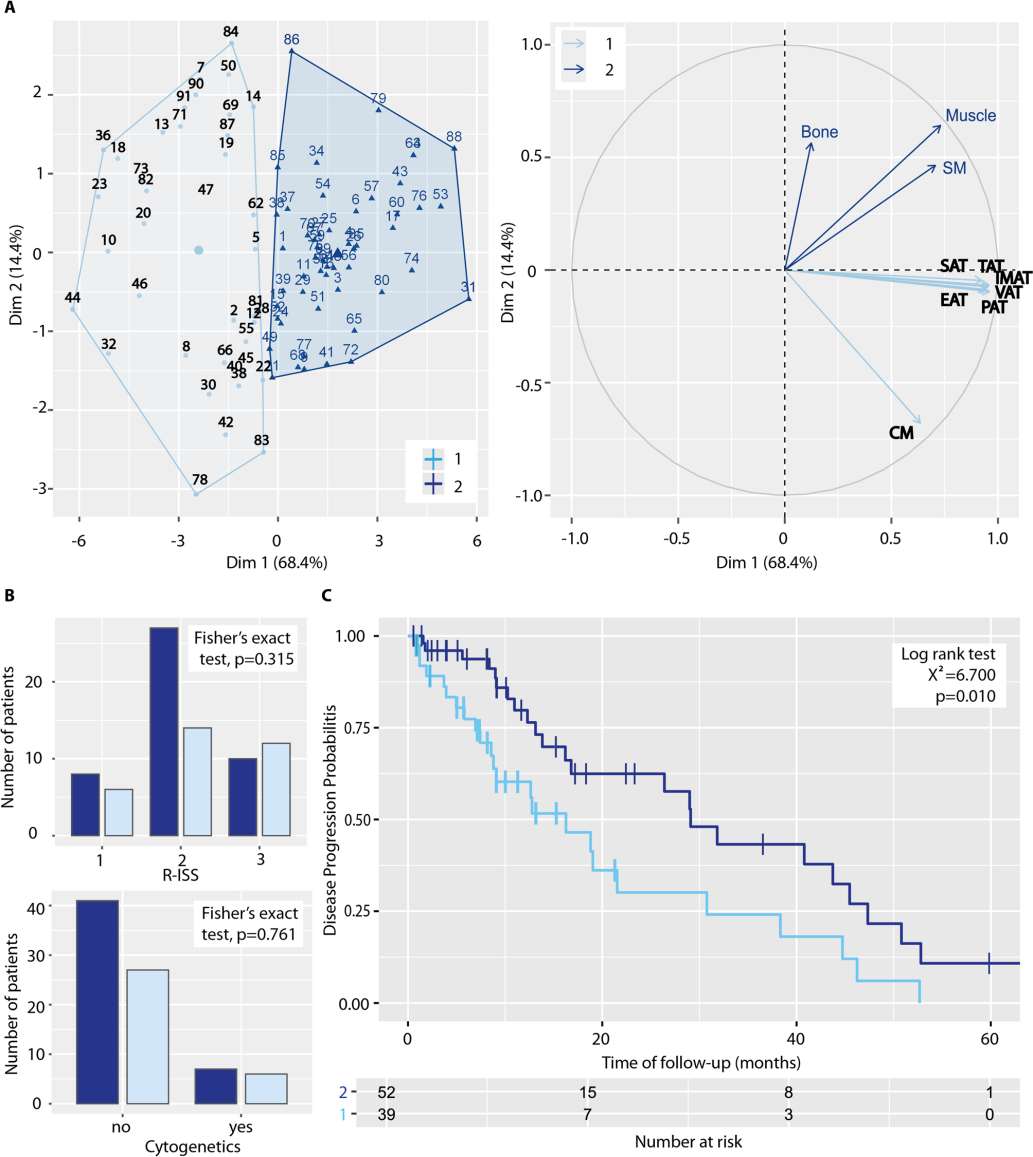
K-means clustering based on normalized BCA parameters, with adjustments for sex and age, identified two distinct clusters. (**A**) The left panel presents a cluster plot dividing patients into two groups based on their body composition. These groups are depicted in light blue (n = 39) and dark blue (n = 52), with each data point representing an individual patient. The right panel features a variables-PCA biplot, illustrating the contributions of various body composition parameters. (**B**) Bar plots display the number of patients with R-ISS scores and high-risk cytogenetics, grouped according to the identified clusters. Fisher’s exact test was used to compare patient counts (R-ISS: *p* = 0.315; high-risk cytogenetics: *p* = 0.761). (**C**) A Kaplan–Meier plot compares disease progression between the two clusters. The log-rank test revealed a significant difference in disease progression probabilities (X^2^ = 6.700, *p* = 0.010).

K-means clustering was performed on normalized BCA parameters, with adjustments for sex and age, resulting in the identification of two distinct patient clusters. The cluster analysis revealed two groups of patients characterized by differences in body composition, as visualized in a cluster plot (n = 39 in light blue and n = 52 in dark blue) in Fig. 3A. A PCA biplot further illustrates the contribution of BCA parameters to the clustering outcome with bone density, muscle volume and SM determining one dimension and the adipose tissue compartments together with CM determining the other dimension. To explore the clinical relevance of these clusters, the distribution of patients with different R-ISS scores and high-risk cytogenetic profiles was analyzed (Fig. 3B). Fisher’s exact test indicated no significant differences between the two clusters regarding the frequencies of these clinical parameters (R-ISS: *p* = 0.315; high-risk cytogenetics: *p* = 0.761). Despite no significant differences in parameters used in clinical practice to predict disease progression and death, a respective comparison between the two clusters revealed a significant difference using log-rank test (X^2^ = 6.700, *p* = 0.010). The corresponding Kaplan–Meier survival analysis is depicted in Fig. 3C. These findings suggest that the identified clusters may reflect distinct disease trajectories.

### Model based patients’ survival prediction

**Fig. 4:**
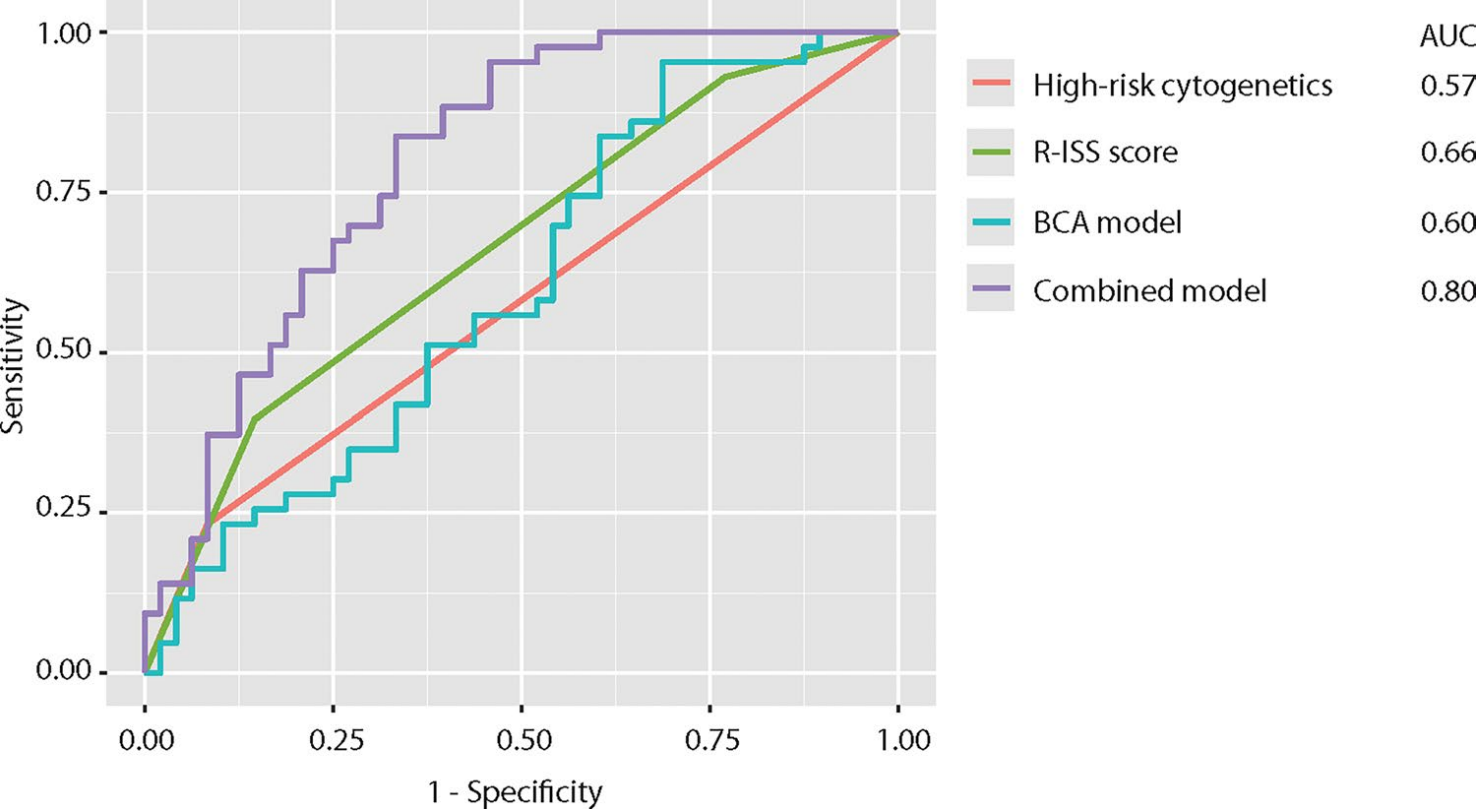
The ROC curves for high-risk cytogenetics, the R-ISS score, the BCA model, and the combined model (both developed using a stepwise model selection algorithm) are shown, comparing their ability to predict progression-free survival versus disease progression in MM patients. The BCA model, derived through the stepwise selection algorithm, included the variables bone density, muscle volume, and SM, with an AIC of 127.37. The combined model, also developed using the stepwise selection algorithm, incorporated muscle volume, TAT, SAT, EAT, CM, ISS, and sex, achieving an AIC of 119.36. The AUC values for all four models are displayed on the right side.

To evaluate the predictive capability for disease progression versus progression-free survival in MM patients, several parameters were analyzed. High-risk cytogenetics and R-ISS scores, both commonly utilized in clinical practice, were included. Additionally, a stepwise model selection process was employed to develop a model based on specific BCA parameters. The resulting BCA model incorporated bone density, muscle volume, and SM, with an AIC of 127.37. A similar approach was undertaken to construct a combined model, integrating clinical parameters such as sex, age, high-risk cytogenetics, and the R-ISS score, along with BCA-derived variables. The generated combined model included the parameters muscle volume, TAT, SAT, EAT, CM, sex and R-ISS. This combined model achieved a superior AIC of 119.36. The ROC curves for high-risk cytogenetics, the R-ISS score, the BCA model, and the combined model are presented in Fig. 4. The AUC values determined from these analyses were 0.57 for high-risk cytogenetics, 0.66 for the R-ISS score, and 0.60 for the BCA model. Notably, the combined model, integrating clinical and BCA parameters, demonstrated the highest predictive performance for disease progression vs. progression-free survival with an AUC of 0.80.

## Discussion

This study illustrates the clinical potential of fully automated AI-based BCA from routinely performed CT-scans of patients with MM. The BCA data allowed for patient clustering and cluster-based differentiation of survival rates. Remarkably, a combined model integrating clinical parameters and BCA data demonstrated superior predictive capability for disease progression compared to models based solely on high-risk cytogenetics or R-ISS.

A major drawback of the traditional manual BCA techniques used so far is their time-consuming and user-dependent nature. The introduction of AI-based fully automated BCA has completely reduced these disadvantages^8^. AI-based analyses have a high level of reproducibility and are not affected by human measurement inaccuracies, associated with redundant tasks, such as segmentations.

CT imaging is a major aspect of the primary diagnostic and follow-up of MM patients. Whole-body low-dose CT-scans are routinely performed at the time of initial diagnosis to detect myeloma manifestations or osteolysis. The fully automated nature of the used BCA approach allows to extract highly reproducible volumetric BCA markers from the patient’s CT data in a timely manner independent and without any additional radiation exposure.

The value of BCA for multiple myeloma remains unclear so far. Results of existing studies are heterogenous and thus limiting the clinical applicability. *Groß *et al*.* found an indirect correlation between high-risk cytogenetics and the amount of visceral adipose tissue^11^. This correlation could not be confirmed in our cohort. In patients with a high-risk cytogenetic profile, we found that the cardiac marker index was significantly higher than in patients without high-risk cytogenetics. The cardiac marker reflects the ratio of epicardial and pericardial fat tissue in relation to the total adipose tissue. So far, the cardiac marker has been associated with poorer outcome or death e.g. in SARS-CoV-2 patients^15^. In a work from *Surov *et al*.* the muscle density was assessed regarding its prognostic value in MM patients undergoing autologous stem cell therapy, but no relevant effect was found^10^. In contrast, in newly diagnosed MM patients, a correlation between myosteatosis and impaired overall survival was identified^12^. Another study including 341 MM patients undergoing^18^ F-FDG PET/CT examinations confirmed the negative predictive effect of low muscle density on clinical outcome parameters, like the patients’ overall survival^16^. All these studies used only a single CT slice from each patient for BCA. In our volumetric analyses, there was no dependency between IMAT, which represents myosteatosis, and clinical outcome parameters observed. But we found that patients with progression or death during follow-up had relevantly lower volumes of total adipose tissue and subcutaneous adipose tissue in contrast to patients of the long-term follow-up group. This finding aligns with the literature, which demonstrates a negative effect of the subcutaneous adipose tissue index on overall survival in a cohort of 56 MM patients^17^. The effects underlying fat loss in cancer patients are diverse^18^. Due to the small cohort size and the study design, no biological correlations can be drawn in this study. However, in larger, prospective studies, the focus should also be put on biological effects that lead to an influence on BCA values.

In our study and in the existing literature, sarcopenia could not be identified as reproducible prognostic value for overall survival^17,19,20^. Possibly, limited cohort sizes, different sarcopenia measurement techniques and heterogenous patient populations limit the proof of sarcopenia as prognostic value in MM patients’ so far. Nevertheless, this is contrary to other chronic diseases and patient cohorts with malignant tumors. E.g. sarcopenia was found to be associated with long-term mortality in patients after coronary artery bypass grafting^5^ and COPD patients^4^. In malignant diseases like hepatocellular carcinoma^3^ and breast cancer^6^, sarcopenia was associated with reduced overall survival rates. Even though a loss of muscle mass, low muscle volume, respectively, is established as a prognostic factor for survival prediction of chronically ill patients, sarcopenia must also be understood as a geriatric symptom^21^.

Multiple myeloma is associated with MM-related bone disease in up to 80% of all cases^22^. A dependency between disease severity and bone mineral density seems to be implicit. While the number of osteolytic bone lesions correlates with the prognosis, no evidence for the bone density as prognostic parameter is given. This is confirmed by our study, as we did not find any difference in bone mineral density in relation to different disease severities. Here, larger prospective study cohorts might offer insights, which may have remained hidden in our work.

Next to the prediction of disease courses based on individual BCA parameters, the clustering of patients beyond established clinical classification systems is a huge potential of BCA. The identification of specific BCA patterns is the basis for selective patient stratification, including the potential to outperform clinical models. In our work, we were able to identify two distinct patient clusters based on their BCA fingerprints. The survival rates of the patient groups identified by using the BCA model differed significantly. Moreover, the high predictive potential of CT-derived BCA in combination with R-ISS and sex enabled a valid patient stratification. These results highlight the potential of BCA to enhance patient stratification and refine prognostic models in MM, especially in combination with already established clinical parameters.

A major limitation of our study is its retrospective nature. The relatively small size of the cohort from a single center limits the informative value. Additionally, a selection bias cannot be excluded, as only patients with routinely performed CT scans were included in the study. With the perspective of BCA`s clinical integration, prospective multi-center data is needed to validate initial findings such as that from our study. Especially, the therapy response and differentiation between BCA values in subgroup analyses are essential to enhance the clinical robustness of the method. In that regard, e.g. a differentiation between transplant-eligible and ineligible patients would be of interest. Furthermore, longer follow-up times will potentially gain deeper insights into the correlation of BCA parameters and clinical outcomes.

In conclusion, CT-derived BCA represents a promising fully automated tool for the assessment of skeletal muscle, bone density and adipose tissue in patients with MM. Its ability to provide quantitative imaging biomarkers with prognostic relevance highlights its potential to improve risk stratification and guide treatment decisions. However, prospective validation and broad technical implementation are essential to establish AI-based CT-derived volumetric BCA in MM care.

## Electronic supplementary material

Below is the link to the electronic supplementary material.

Supplementary Material 1

Supplementary Material 2

Supplementary Material 3

## Data Availability

The data that support the findings of this study are available upon request from the corresponding author.
