# Supplementary material for: AI-based body composition analysis of CT data has the potential to predict disease course in patients with multiple myeloma
Source: Sci Rep. 2025 Jul 21;15:26455. doi: 10.1038/s41598-025-11560-3 (PMC12280154; doi:10.1038/s41598-025-11560-3)
Supplement: Supplementary file 1 — Supplementary Material 1 [file 41598_2025_11560_MOESM1_ESM.docx]

**Supplementary Figure 1.** (**A**) **Bone density, EAT and CM exhibit significant correlations with age.** The figure displays a Spearman correlation matrix illustrating the relationship between age and BCA parameters. The Spearman r correlation coefficient is shown at the top of each box, while the corresponding p-value is displayed below. P-values < 0.05 are highlighted in light red, and those < 0.001 are marked in dark red. (**B**) **Muscle volume, VAT, PAT, EAT, CM and SM vary significantly between sexes.** Males are represented in green, while females are shown in orange. The results of the Mann-Whitney U test are provided beneath each corresponding plot.
