# Supplementary figures and images for: AI-based body composition analysis of CT data has the potential to predict disease course in patients with multiple myeloma
Source: Sci Rep. 2025 Jul 21;15:26455. doi: 10.1038/s41598-025-11560-3 (PMC12280154; doi:10.1038/s41598-025-11560-3)

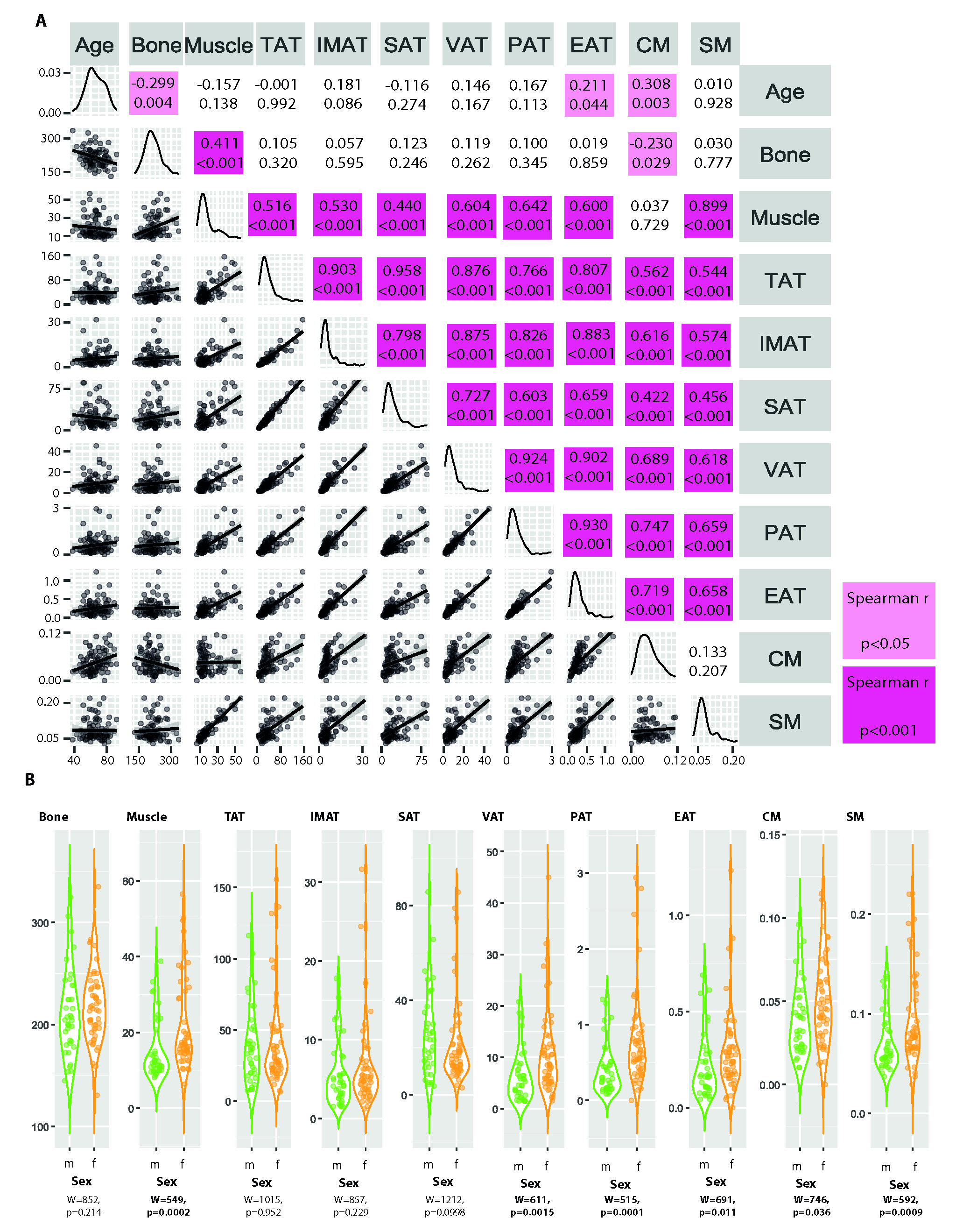

Supplement: Supplementary file 2 — Supplementary Material 2 [file 41598_2025_11560_MOESM2_ESM.tif]
