# Supplementary material for: AI-based body composition analysis of CT data has the potential to predict disease course in patients with multiple myeloma
Source: Sci Rep. 2025 Jul 21;15:26455. doi: 10.1038/s41598-025-11560-3 (PMC12280154; doi:10.1038/s41598-025-11560-3)
Supplement: Supplementary file 3 — Supplementary Material 3 [file 41598_2025_11560_MOESM3_ESM.docx]

# Supplementary Table 1: Distribution of First-Line Therapies and Autologous Stem Cell Transplantation in the Study Cohort (n = 91)

|  |  |
| --- | --- |
| **First-line Therapy** | **n = 91 (100%)** |
| VD | 19 (20.9 %) |
| VCD | 18 (18.7 %) |
| Dara-VTD | 14 (15.4 %) |
| VRD | 9 (9.9 %) |
| Dara-RD | 7 (7,7 %) |
| Dara-VD | 4 (4.4 %) |
| Other | 20 (22,0 %) |
| **Autologous Stem Cell Transplantation** |  |
| Performed | 39 (42,9 %) |
| Not performed | 52 (57.1 %) |

**VD** = Bortezomib, Dexamethasone; **VCD** = Bortezomib, Cyclophosphamide,
Dexamethasone; **Dara-VTD** = Daratumumab, Bortezomib, Thalidomide, Dexamethasone; **VRd** = Bortezomib, Lenalidomide, Dexamethasone; **Dara-RD =** Daratumumab, Lenalidomide, Dexamethasone**; Dara-VD** = Daratumumab, Bortezomib, Dexamethasone
